# Berberine—A Promising Therapeutic Approach to Polycystic Ovary Syndrome in Infertile/Pregnant Women

**DOI:** 10.3390/life13010125

**Published:** 2023-01-02

**Authors:** Oana-Maria Ionescu, Francesca Frincu, Andra Mehedintu, Mihaela Plotogea, Monica Cirstoiu, Aida Petca, Valentin Varlas, Claudia Mehedintu

**Affiliations:** 1 Faculty of Medicine “Carol Davila”, University of Medicine and Pharmacy Bucharest, 050474 Bucharest, Romania; 2 Department of Obstetrics and Gynecology, “Nicolae Malaxa” Clinical Hospital, 022441 Bucharest, Romania

**Keywords:** infertility, pregnancy, polycystic ovary syndrome, insulin resistance, berberine

## Abstract

Polycystic ovary syndrome (PCOS) is a disorder with an unknown etiology that features a wide range of endocrine and metabolic abnormalities that hamper fertility. PCOS women experience difficulties getting pregnant, and if pregnant, they are prone to miscarriage, gestational diabetes, pregnancy-induced hypertension and preeclampsia, high fetal morbidity, and perinatal mortality. Insulin, the pancreatic hormone best known for its important role in glucose metabolism, has an underrated position in reproduction. PCOS women who have associated insulin resistance (with consequent hyperinsulinemia) have fertility issues and adverse pregnancy outcomes. Lowering the endogen insulin levels and insulin resistance appears to be a target to improve fertility and pregnancy outcomes in those women. Berberine is an alkaloid with a high concentration in various medicinal herbs that exhibits a hypoglycaemic effect alongside a broad range of other therapeutic activities. Its medical benefits may stand up for treating different conditions, including diabetes mellitus. So far, a small number of pharmacological/clinical trials available in the English language draw attention towards the good results of berberine’s use in PCOS women with insulin resistance for improving fertility and pregnancy outcomes. Our study aims to uncover how berberine can counteract the negative effect of insulin resistance in PCOS women and improve fertility and pregnancy outcomes.

## 1. Introduction

Polycystic ovary syndrome (PCOS) is a heterogeneous disorder whose etiology and pathogenesis is supposedly the consequence of the interaction between genetic and environmental factors, both in intrauterine and postnatal life [1,2]. PCOS does not only affect women of reproductive age (with a prevalence of 9% to 18% depending on the diagnostic criteria) [3,4] but also adolescents and postmenopausal women [2].

The expression of polycystic ovary syndrome cannot thoroughly comprise the complexity of the endocrine and metabolic abnormalities encountered in this syndrome.

The prototype of PCOS women has impaired reproductive function, marked insulin resistance (IR), and dyslipidemia [5,6]. Additional to infertility, women with PCOS present a higher risk of developing obesity, cardiovascular disease, diabetes mellitus, endometrial, ovarian, and breast cancer, or mood disorders, such as depression and anxiety [7]. Regardless of which PCOS definition is used, 60% of PCOS women and 30% of their offspring’s offspring are subject to perinatal and neonatal complications [8]. Pregnant PCOS women are prone to miscarriage, to develop gestational diabetes mellitus—GDM (3-fold increased risk), pregnancy-induced hypertension and preeclampsia (3–4-fold increased risk), fetal macrosomia, or small-for-gestational-age infants and high perinatal mortality [5,6,9]. PCOS pregnant women with hyperandrogenism (a consequence of hyperinsulinemia) have a high risk of developing pregnancy hypertensive disorders due to testosterone’s ability to interfere with trophoblast invasion and placenta morphology and function [9].

Insulin plays a crucial role in metabolism, cell growth, and reproductive function [10]. The main metabolic activity of insulin is to achieve and maintain normal ranges of blood glucose levels [11]. Insulin resistance, a condition encountered in 85% of PCOS women regardless of their BMI [12,13], represents the need for higher-than-normal quantities of insulin to maintain normal blood glucose. In PCOS women pancreatic β cell activity augments up to the point when the pancreatic cells’ production of insulin depletes, and PCOS women develop glucose intolerance or type II diabetes [11]. Through complex mechanisms, both insulin resistance and hyperinsulinemia hamper fertility and lower pregnancy outcomes [10,12]. In PCOS women, the close relationship between hyperinsulinemia and hyperandrogenism [4,12,14,15] creates a toxic environment that hampers fertility and disturbs the course of pregnancy [16]. Knowing that insulin resistance and its subsidiary hyperinsulinemia/hyperandrogenism are limitational features in PCOS women when speaking of fertility and pregnancy outcomes, it seems only natural to want to lower the endogen insulin levels and/or insulin resistance to improve fertility and pregnancy outcomes in those women. PCOS women can decrease insulin resistance by losing weight (if obese) or using insulin-sensitizing agents [12]. There are many insulin-sensitizing agents that can be used in everyday practice. Among these, berberine (BBR) seems to be a powerful tool. 

## 2. Materials and Methods

We searched internet databases for any available information concerning alternative treatments for insulin resistance in PCOS infertile/pregnant women. Our search found reports about berberine, a natural compound with a wide range of therapeutic activities including the ability to improve fertility and pregnancy outcomes in PCOS women. Different electronic databases were searched (PubMed, SciELO, ReaserchGate, Hindawi, PloS One, and Science Direct) to identify studies related to berberine’s effect in infertile/pregnant PCOS women with insulin resistance. The reference lists of the included studies were also screened for additional literature. We used “MeSH” (PubMed) terms for our search: berberine, polycystic ovary syndrome, insulin resistance, and fertility/pregnancy. The research was not restricted to a particular study type to be as comprehensive as possible. We focused only on those articles published in the English language that contained in the main text information about the relationship between berberine’s effect on PCOS infertile/pregnant women and excluded duplicate studies. Our search included all studies from 2000 to the present day. 

## 3. Results

### 3.1. Natural Sources of Berberine and Its Uses

Although we found numerous studies concerning the overall benefits of berberine, our search criteria pointed out that there is little information available in the English language concerning berberine’s ability to improve fertility and pregnancy outcomes in PCOS women.

**Figure 1:**
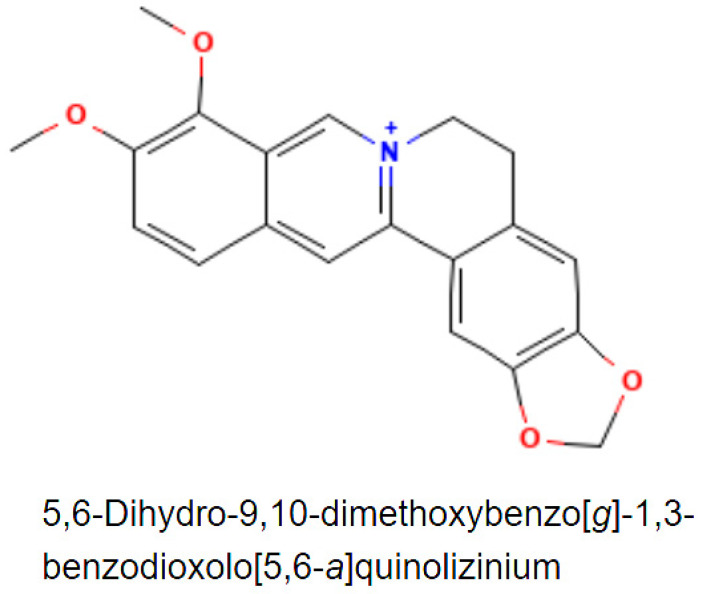
Berberine (C20H18NO4+) 2D chemical structure [19,20].

Berberine is a quaternary ammonium salt (Figure 1) from the protoberberine group of isoquinoline alkaloids with a molar mass of 336.36122 g/mol [12,17,18,19].

Due to its deep yellow and yellow fluorescent characteristics berberine was initially used as a dye for wood, wool, and leather [21,22]. The oldest evidence of the medicinal properties of Berberis plants dates back almost 3000 years in ancient Assyria where barberry fruits (from Berberis vulgaris) were used as a blood-purifying agent [23]. Since then, there have been many reports of the extensive use of the leaves, roots, rhizomes, fruits, and stem bark of plants rich in berberine, particularly in Ayurvedic and traditional Chinese medicine, which exploited the beneficial effects of berberine [19,24]. Berberine is widely distributed in all parts of plants, nevertheless, bark and roots are richer in berberine compared with other plant parts, and its concentration is highest in the summer harvesting period [19,25], except for B. aristata where the berberine concentration is higher in the winter season [24]. Furthermore, the maximum concentration of berberine is obtained from plants growing at low altitudes compared with higher altitude-growing plants [24]. From the great Berberidaceae family, the genus Berberis comprises ∼450–500 species, which represent the most widely distributed natural source of berberine [23]. The bark of B. vulgaris contains more than 8% of alkaloids, berberine being the major alkaloid (about 5%) [24]. Berberine has also been detected, isolated, and quantified from other various plant families and genera including *Annonaceae* (*Annickia*, *Coelocline*, *Rollinia*, and *Xylopia*), *Menispermaceae* (*Tinospora*), *Papaveraceae* (*Argemone*, *Bocconia*, *Chelidonium*, *Corydalis*, *Eschscholzia*, *Glaucium*, *Hunnemannia*, *Macleaya*, *Papaver*, and *Sanguinaria*), *Ranunculaceae* (*Coptis*, *Hydrastis*, and *Xanthorhiza*), and *Rutaceae* (*Evodia*, *Phellodendron*, and *Zanthoxylum*)—Table 1 [12,17,19,24,26].

Berberine’s medical properties are still under investigation due to its ability to interact with various biological targets involved in the pathogenesis of multiple diseases [27]. Berberine’s wide range of therapeutic activities include antimicrobial, antiviral, and cholagogue activity, its cytotoxic effect, its use as an antiemetic, antipyretic, antipruritic, antioxidant, anti-inflammatory, antiarrhythmic, antinociceptive, anticholinergic cardiotonic, a contraceptive, a hypotensive, a hypoglycemic, a neuroprotective, and it being efficient in treating different diseases (diabetes, cardiovascular diseases, cancer, depression, inflammatory diseases, cholecystitis, cholelithiasis, jaundice, dysentery, leishmaniasis, malaria, and gall stones) [17,24,28,29,30,31,32,33,34,35,36]. Today, the hydrochloride salt of berberine is listed as an oral antibacterial agent in Pharmacopoeia of the People’s Republic of China [37]. In everyday life, we use food supplements containing berberine such as turmeric (a yellow powder extracted from B. aristate) to spice up our food.

### 3.2. Berberine Pharmacokinetics

Pharmacokinetics studies (conducted mainly in a rat model) targeted the absorption, tissue distribution, and elimination of berberine and showed that despite its poor intestinal absorption and low oral bioavailability (less than 1%) [17,28,33], berberine is found in the rat’s liver, kidneys, muscle, lungs, brain, heart, and pancreas, with concentration levels that equal or exceed plasma levels after 4-h administration [29,38]. Low plasma concentrations of berberine in vivo can be explained by its conversion to an ionized form in physiological conditions and self-aggregation in low pH conditions thus decreasing its solubility and its permeability in the gastrointestinal tract [19,33]. The poor water solubility of berberine caused by the rigid planer structure and quaternary ammonium unit always leads to very low absorption efficiency in the gastrointestinal tract [39]. Other barriers for low oral bioavailability are hepatobiliary re-extraction, P-glycoprotein (P-GP) mediated efflux, and metabolization by CYP2D6 and CYP3A4 in the intestine (by gut flora) which transform berberine into metabolites—berberrubine, demethyleneberberine, and jatrorrhizine—whose bioavailability is also low [17,19,29,33]. 

Despite its low bioavailability, berberine has a wide range of biological activities. The contradiction between the definitive curative effect of berberine and its extremely low plasma concentration raised the hypothesis that berberine’s metabolites may also contribute to BBR’s metabolic activity. This paradox was solved by extensive investigations that gained evidence of the simultaneous existence and exercise of metabolic activities by both BBR and BBR’s metabolites [40]. Studies showed that after 0.5 h of oral intake, metabolites of berberine are found in the liver, thus making the liver the primary site for the metabolic transformation of berberine [19,29,41]. Under the action of five cytochrome P450 (CYP) enzymes (CYP2D6, CYP1A2, 3A4, 2E1, CYP2C19), berberine undergoes two types of metabolic transformation: phase I—demethylation and phase II—glucuronidation, leading to berberrubine, demethyleneberberine, jatrorrhizine, and thalifendine (its four major metabolites), and their respective glucuronide conjugates [19,28,29,41,42]. We know now that jatrorrhizine, columbamine, and palmatine (phase I metabolites) have similar lipid-lowering and anti-ulcerative colitis effects with BBR, and demethyleneberine (another phase I metabolite) was proven to show anti-inflammatory and hepatoprotective effects, while berberineberberrubine-9-O-β-D-glucuronide (phase II metabolite) showed a glucose-lowering effect with a higher potency even than BBR [42]. 

Altering the chemical structure of berberine is a strategy to provide more novel active antidiabetic molecules in terms of bioavailability [27]. Structure-activity relationship investigation reveals that the methylenedioxy group and quaternary ammonium unit in berberine are two key structural components related to its anti-diabetic activity [43]. For example, when the methylenedioxy group is removed or the quaternary ammonium salt reduces to a tertiary amine it results in a significant decrease in the anti-diabetic effect of berberine [39], while some C-8,13-substituted berberine derivatives have significant glucose-lowering properties, and the 9-OH or 10-OH pseudo-berberines also exhibited high anti-diabetic activity [39,43]. The 9-O-position modification of berberine (9-O-hydrophilic and hydrophobic modification) can both improve its bioavailability and enhance antidiabetic activity [27,39,43]. Other structural alterations of the BBR formula that have glucose-lowering effects are the introduction of free carboxyl groups at tetrahydro berberine moiety at N-7 or C-6. An acetic acid group attached at the C-6 position of protoberberine exhibits higher potency than carboxyl substituent. The attachment of OCH3 groups on ring-D of tetrahydro berberine at C-10 and C-11 positions imparted excellent hypoglycemic effects [27]. The alteration of the original BBR chemical formula proved to be unstable under mild conditions which are easily degraded into berberine and related analogs in vitro. Studies on 9-O-glucosyl-berberine (berberine metabolite with glucose-lowering effect) revealed that the compound has higher bioavailability and thus higher hypoglycemic effect when its water solubility increases (via synthesis of a hydrophilic compound) [27,43]. The conjugation of monosaccharides with berberine (mannose) has significantly lower cytotoxicity than berberine and exhibits high anti-diabetic activity at both high and low drug concentrations, thereby indicating its potential application for the development of novel anti-diabetic drugs [43]. Furthermore, animal studies on disaccharide moiety conjugated to berberine are promising in their aim to deliver a molecule with higher anti-diabetic activity and a much more stable chemical structure in normal conditions [27,39].

Pharmaceutical excipients are currently under investigation to improve intestinal absorption and oral bioavailability. The intention is to interfere with P-GP-mediated efflux or intestinal metabolism and thus increase berberine plasma levels (Silybum marianum extract, sodium caprate, chitosan, oral microemulsion formulation of BBR, oral microemulsion formulation of BBR, self-micro emulsifying drug delivery system of BBR) [44,45]. Furthermore, oral bioavailability is improved when berberine is delivered as berberine organic acid salts (fumarate or succinate) than as berberine hydrochloride [38]. Nano-sized dosage forms and nanocrystal dosage forms emerge as important solutions for the bioavailability enhancement of a low solubility and dissolution rate such as BBR, with studies showing higher plasma levels of BBR after oral intake of these delivery systems [42].

### 3.3. Berberine’s Hypoglycemic Effect

As mentioned before, berberine and its metabolites exhibit various metabolic activities, its hypoglycaemic effect being exploited today as an alternative therapy in diabetes. It is very interesting to see the intricate pathways via which berberine exerts its hypoglycaemic effect. It alleviates insulin resistance, promotes insulin secretion, promotes glucose uptake, induces glycolysis, and inhibits gluconeogenesis in the liver [21,29,38,46]. Understanding the way berberine acts and lowers endogen insulin levels, thereby reducing hyperinsulinemia side effects, can be exploited in clinical practice by adding BBR in everyday therapy to alleviate PCOS pregnant and nonpregnant women’s hyperinsulinemia side effects

#### 3.3.1. Berberine Alleviates Insulin Resistance

The insulin receptor (InsR) is a vital factor in the insulin signaling pathway. BBR increases the activity of InsR mRNA and upregulates protein kinase C-dependent InsR expression, thereby effectively increasing glucose utilization and improving IR. Simultaneously, BBR upregulates InsR gene expression in muscle and in the liver in a protein kinase D- (PKD-) dependent manner, which contributed to promoting insulin sensitivity. [38,47,48].

Another mechanism by which BBR acts as an insulin-sensitizing agent is via the AMP-dependent protein kinase (AMPK) pathway. AMP-dependent protein kinase is crucial for the body’s systemic energy homeostasis [38]. BBR enhances insulin sensitivity to maintain glucose homeostasis through AMPK activation in muscle and adipose tissue via two main mechanisms: BBR inhibits respiratory complex I of the mitochondrion, thereby stimulating the AMPK activity, and BBR upregulates the expression of sirtuin 1(SIRT1) in adipose tissue so that the AMPK pathway can be activated [38,49]. Furthermore, BBR interferes with glycolipid metabolism in muscle tissues by promoting the expression of insulin-sensitive glucose transporters (GLUT4), alongside the peroxisome proliferator-activated receptors a (PPARa) and, in the fat tissue, BBR regulates the expression of positive transcription elongation factor b [17,29,50,51]. In vitro exposure of skeletal muscle cells to berberine increases the protein expression of PPARa and the mRNA expression of Glut-4, thus upregulating PPARa and Glut-4 expression, improving cellular glucose uptake, and lowering glucose plasma levels [17,29,50]. Fibroblast growth factor 21 (FGF21) is a hormone derived from the liver with important roles in body metabolism. In the animal model, BBR regulates glucose metabolism and increases insulin sensitivity by inducing FGF21 production and secretion and promoting white fat browning [38]. Other mechanisms of increasing insulin sensitivity and alleviating IR include BBR’s activation of the phosphatidylinositol 3-kinase (PI3K) pathway, which in turn raises GLUT4; BBR’s inhibition of the protein tyrosine phosphatase 1B activity in adipocytes in a dose-dependent manner; BBR’s inhibition of the intestinal α-glucosidase (thus reducing the intestinal absorption of monosaccharides); the inhibition of the lipopolysaccharide (LPS)/toll-like receptor 4 (TLR4)/tumor necrosis factor (TNF)-α signaling pathway (preventing insulin resistance worsening); and BBR’s alleviation of the IR via regulation on the protein phosphatase, Mg^2+^/Mn^2+^-dependent 1B (PPM1B) signaling pathway [27,35,38,47,48,51,52,53,54]. 

#### 3.3.2. Berberine Promotes Insulin Secretion

Berberine promotes insulin secretion in the following ways: BBR stimulates insulin secretion by improving PARP-1 protein expression and pancreatic β-cells proliferation; BBR increases the secretion and synthesis of glucagon-like peptide-1 (GLP-1)—an intestinal hormone implicated in stimulating insulin secretion, promoting pancreatic β-cells proliferation, and regulating glucose metabolism when blood sugar is elevated; BBR enhances insulin secretion in diabetic mice and rat insulinoma cell lines via the activation of the uncoupling protein 2 (UCP2—protein with the function of regulating glucose-stimulated insulin secretion GSIS) and AMPK pathways; and in mice BBR causes pancreatic β-cell proliferation and GSIS through an enhanced insulin/insulin-like growth factor-1 signaling cascade and pathways related to hepatocyte nuclear factor 4α and glucokinase [38,55,56,57].

#### 3.3.3. Berberine Promotes Glucose Uptake

Glucose transporters (GLUT) are essential for glucose metabolism. GLUT 1 is found in all human tissues, while GLUT4 is mainly expressed in insulin-sensitive tissues such as skeletal muscle, fat cells, and myocardium [38]. BBR can activate GLUT1 and upregulate GLUT1 expression level through the AMPK pathway [55,57] and as mentioned before, BBR increases GLUT4 expression and translocation activity by dual regulation of the PI3K/AKT and MAPK pathway [54], thereby enhancing the glucose uptake and elevating the glucose availability by tissues and cells of the body [38]. 

#### 3.3.4. Berberine Reduces Glucose Intestinal Absorption

Inhibiting 𝛼-Glucosidase (the intestinal enzyme responsible for digesting carbohydrates and converting them into monosaccharides) makes the digestion and absorption of carbohydrates suppressed. In the rat duodenum, BBR suppresses the expression of 𝛼-Glucosidase and downregulates the β-glucuronidase, thus decreasing glucose transport through the intestinal epithelium [35,58].

#### 3.3.5. BBR Induces Glycolysis and Inhibits Gluconeogenesis in the Liver

Berberine stimulates glycolysis in two ways: first, BBR activates the AMPK pathway by inhibiting mitochondrial glucose oxygenation that leads to an increase in AMP/ATP ratio, and second, BBR can inhibit respiratory complex I of the mitochondria, thereby inhibiting ATP synthesis and enhancing glycolysis (an AMPK independent pathway) [59,60].

Glucose-6-phosphatase (G6 Pase) and phosphoenolpyruvate carboxykinase (PEPCK) were two key rate-limiting enzymes that regulated hepatic gluconeogenesis. [38,55]. BBR inhibits mitochondrial function, and the expression of PEPCK and G6 Pase genes, thereby inhibiting gluconeogenesis [38,61]. In addition, BBR inhibited the expression of PEPCK 1 (secondary to SIRT 3 inhibition) and G6Pase by upregulating the protein expression of the liver kinase B1 (LKB1) and AMPK and inhibiting the translocation of the cAMP response element-binding protein (CREB)-regulated transcription co-activator 2 (TORC2) into the cell nucleus [55,61]. BBR suppresses the HNF-4α miR122 pathway in type 2 diabetic mice, which attenuates gluconeogenesis and lipid metabolism disorder [38].

### 3.4. Berberine Improves Fertility and Pregnancy Outcome in PCOS Women

Insulin resistance and its subsidiary hyperinsulinemia/hyperandrogenism are limitational features in PCOS women when speaking of fertility and pregnancy outcomes, and berberine exhibits a wide range of metabolic effects that can be exploited in everyday practice in order to achieve higher pregnancy rates and lower pregnancy complications in those women.

Compared with healthy women, PCOS women have an infertility rate 15 times greater and an early pregnancy loss rate three times greater [16]. Moreover, PCOS women with insulin resistance are predisposed to develop diabetes mellitus in both pregnant and non-pregnant states. Through intricate mechanisms, insulin resistance with subsidiary hyperinsulinemia/hyperandrogenism [4,12,14,15] in PCOS women leads to a disturbance in granulosa cell differentiation and arrests follicle growth [10,62], alters the granulosa cell-oocyte interactions, and damages oocyte maturation [63]. At the endometrial level, the same promotes abnormally high levels of androgen receptors and the failure to down-regulate estrogen receptor α during the window of implantation, resulting in low pregnancy rates, and higher early pregnancy abortion rates [15,64]. Furthermore, the hyperandrogenic–hyperinsulinaemic environment in pregnant PCOS women promotes prothrombotic and profibrotic activities which have a pro-inflammatory effect and potentiates vascular alterations, which ultimately leads to adverse placental changes, alterations in the uterine artery flow, and gestational hypertension that will lead to difficulties in embryo implantation, increasing the risk of miscarriage or influencing fetal growth [16]. In PCOS animals exhibiting insulin resistance, BBR improves endometrial receptivity via modulating the endometrial implantation genes (LPAR3, αvβ3, and HOXA11), although the exact mechanism is still under investigation [65,66].

Our search of internet databases for any available information concerning’ berberine’s ability to improve fertility and pregnancy outcomes in PCOS women with insulin resistance pointed out the scarce information available for English language readers concerning our pursuit. We found evidence that berberine intake may improve menstrual patterns in PCOS infertile women with chronic anovulation up to the point of regaining regular menses and ovulation second to an insulin resistance decrease [67]. In a different study berberine proved to be efficient in improving IVF pregnancy rates and metabolic parameters (fasting plasma glucose, fasting insulin, and insulin resistance), lowering the risk of ovarian hyperstimulation syndrome in a similar manner to metformin, and allowing the reduction of the total FSH doses per IVF cycle. It was also found to surpass metformin when speaking of live birth rates, and berberine surpasses placebo medication in all the proposed objectives, showing no additional advantages to metformin when comparing the total number of days of ovarian stimulation, the mean number of oocytes collected, the diploid fertilization rate, and the mean number of embryos transferred [12]. In PCOS women, berberine alone has no superior efficacy to letrozole alone/a letrozole-berberine combination in terms of cumulative live birth rate, ovulation rate, conception rate, clinical pregnancy rate, and pregnancy loss rates before 20 weeks of gestation. On the contrary, conception, pregnancy, and ovulation rates are significantly lower when using berberine alone compared with letrozole [68,69,70]. The beneficial use of berberine to lower insulin resistance and hypoglycemia in pregnant women with GDM is advocated by the existing proof about the way berberine lowers the levels and determines a higher degree of methylation of the hypoxia-inducible factor-3α gene (HIF3A), the gene related to insulin sensitivity in adipose tissue [71].

### 3.5. Berberine Intake Side Effects

The side effects of BBR intake are not null. In animal studies and clinical trials, BBR has shown low toxicity [55]. In humans, the dosage of BBR used to treat different metabolic diseases ranges from 0.4 g/day to 1.5 g/day [55,72]. The adverse effects reported are mild, transient, and sporadic: anorexia/loss of appetite, abdominal pain, nausea, vomiting, diarrhoea, constipation, and flatulence [73] However, the side effects of BBR are related to its dosage and route of administration [55].

Berberine has poor oral bioavailability, and intravenous injection can increase the drug plasma concentration; although this administration route is appealing, the method has more side effects than the oral route (respiratory arrest, hypotension) [38].

Oocyte growth, maturation, and viability end embryo post-implantation development are sensitive to environmental factors such as oxygen concentration or glucose content. Despite its ability to prevent or attenuate H_2_O_2_-induced oxidative injury and apoptosis in smooth muscle cells, motor neuron-like cells, or endothelial and mesangial cells, berberine has amphiphilic effects, exerting both beneficial and harmful effects on oocyte development in a dose-dependent manner. Thus, berberine’s harmful effects on pre-implantation and post-implantation embryonic development are due to the activation of reactive oxygen species (ROS)-mediated apoptotic cascades (animal model) at high doses (5 mg/Kg), while small doses (1 mg/kg) enhance oocyte maturation, IVF rate, and early-stage embryo development after fertilization [74,75,76].

One limitation is the little evidence, though encouraging, regarding the use of berberine and its positive effects, compared with metformin. Although our study is a mere review, we hope our work may inspire others to elaborate on the research further.

A meta-analysis on the ovulation, pregnancy, and live birth rates after using berberine would be challenging since there are few randomized control trials. Further studies are needed to evaluate the effect of BBR in improving fertility and pregnancy outcomes in PCOS women.

## 4. Conclusions

Our findings show that there is an enormous amount of information on the internet concerning PCOS metabolic and endocrine effects that shadow female fertility and the complications that may occur during pregnancy. Still, little is known about berberine being used to amend PCOS women’s situation. PCOS is still an undeciphered endocrine and metabolic disorder closely related to insulin resistance. Since PCOS women experience insulin resistance effects throughout their entire lives, it is of paramount importance to properly control the disease. This paper aims to draw attention to the severity of hyperinsulinemia complications (because of insulin resistance) in infertile or pregnant PCOS women in the absence of appropriate treatment. Insulin sensitizers control metabolic abnormalities and improve reproductive outcomes by improving insulin resistance in PCOS women. There is encouraging evidence of the superior effect of berberine alone vs. placebo/no regimen regarding the live birth rate, ovulation rate, androgen levels, decreasing fasting plasma glucose, and insulin levels, as well as the lowering of insulin resistance. 

## Figures and Tables

**Table 1:** List of plants containing berberine [24,26].

Family	Scientific Name of the Plant	Common Name of the Plant	Plants Used in Gynecology Condition/Diabetes
*Annonaceae*	*Annickia chlorantha*		Diabetes; promote conception
	*Annickia Pilosa*		
	*Annickia polycarpa*		
	*Rollinia mucosa*		
	*Xylopia polycarpa*		
*Berberidaceae*	*Berberis actinacantha*		
	*Berberis aquifolium*	*Oregon grape*	Sore womb following childbirth and/or menstruation
	*Berberis aristata*	*Tree turmeric*	Menorrhagia
	*Berberis asiatica*	*Chutro*	Diabetes
	*Berberis buxifolia*		
	*Berberis chitria*		
	*Berberis congestiflora*	*Michay*	
	*Berberis croatica*	*Croatian barberry*	
	*Berberis darwinii*		
	*Berberis empetrifolia*		
	*Berberis floribunda*	*Nepal barberry*	
	*Berberis integerrima*		Diabetes
	*Berberis jaeschkeana*		
	*Berberis koreana*		
	*Berberis leschenaultia*		Treatment of complications during post-natal period
	*Berberis libanotica*		
	*Berberis lyceum*	*Boxthorn barberry*	Diabetes
	*Berberis microphylla*		
	*Berberis oblonga*		
	*Berberis petiolaris*	*Chochar*	
	*Berberis pseudumbellata*		Oxytocic effect
	*Berberis thunbergia*		
	*Berberis tinctoria*	*Nilgiri barberry*	
	*Berberis umbellate*		
	*Berberis vulgaris*	*Berberry*	
	*Caulophyllum thalictroides*		Inducer of menstruation
	*Jeffersonia diphylla*		
	*Mahonia fortune*		
	*Mahonia napaulensis*		
	*Nandina domestica*		
	*Sinopodophyllum hexandrum*		Regulate menstruation, treat amenorrhea, difficult labor and retention of dead fetus or placenta
*Menispermaceae*	*Tinospora sinensis*	*Heart leaves moonseed*	Anti-diabetic effect
*Papaveraceae*	*Argemone albiflora*		
	*Argemone Mexicana*	*Prickly poppy*	
	*Argemone platyceras*		
	*Bocconia frutescens*		
	*Chelidonium majus*		
	*Corydalis solida*		
	*Corydalis turtschaninovii*		Dysmenorrhea
	*Eschscholzia californica*	*Californian poppy*	Suppress the milk in lactating women
	*Glaucium corniculatum*		
	*Macleaya cordata*		
	*Macleaya macrocarpa*		
	*Papaver dubium*	*Long head poppy*	
	*Papaver rhoeas*		
	*Papaver hybridum*	*Poppy*	
*Ranunculaceae*	*Coptis chinensis*	*Chinese goldthread*	Diabetes
	*Coptis japonica*	*Japanese goldthread*	
	*Coptis teeta*		
	*Hydrastis canadensis*	*Goldenseal*	Vaginal disorders
	*Xanthorhiza simplicissima*	*Yellow root*	
*Rutaceae*	*Phellodendron amurense*	*Amur cork tree*	Vaginal infections (with Trichomonas vaginalis)
	*Zanthoxylum monophylum*		

## Data Availability

Not applicable.
